# Efficient large-scale land cover change detection using Google Earth Engine: Climate-driven vegetation dynamics in Asian drylands (2001–2022)

**DOI:** 10.1371/journal.pone.0344835

**Published:** 2026-04-01

**Authors:** Jianfeng Wu, Shengtao Wei, Haichao Hao, Meng Chen, Sadaf Ismail

**Affiliations:** 1 Guizhou Provincial Key Laboratory of Geographic State Monitoring of Watershed, School of Geography and Resources, Guizhou Education University, Guiyang, China; 2 Key Laboratory of Geographic Information Science (Ministry of Education), School of Geographic Sciences, East China Normal University, Shanghai, China; 3 School of Life Science, Shanxi University, Taiyuan, China; 4 Graduate School of Advanced Integrated Studies in Human Survivability, Kyoto University, Kyoto, Japan; Escola de Engenharia de São Carlos da Universidade de São Paulo: Universidade de Sao Paulo Escola de Engenharia de Sao Carlos, BRAZIL

## Abstract

Monitoring land cover dynamics and understanding vegetation responses to climate change are critical for ecological assessment and management in dryland regions. This study systematically analyzes land cover dynamics, vegetation type transitions, and their climatic drivers across Asian drylands from 2001 to 2022 by integrating MODIS land cover data, TerraClimate climate reanalysis datasets, and the Google Earth Engine (GEE) platform. Using a unified framework that combines land cover dynamic indices, transition probability and transfer matrix analyses, and climate attribution, we quantify spatiotemporal change patterns and identify dominant vegetation transition pathways. The results reveal pronounced land cover changes across Asian drylands over the past two decades, characterized by expansions of grasslands (GRA), savannas (SAV), croplands (CRO), and water, snow, and ice (WSI), alongside contractions of shrublands (SH), mixed forests (MF), permanent wetlands (WET), and barren land (BAR). Land cover transition analysis indicates that the most prominent conversion pathways are from barren land to grasslands and from grasslands to croplands, reflecting the combined influences of climate variability and land use processes. Climate attribution analyses further demonstrate that vegetation dynamics across different stability zones exhibit distinct responses to long-term climate trends, with increasing maximum temperature, soil moisture, and vapor-related variables, together with declining precipitation, drought indices, and surface radiation, jointly shaping vegetation persistence, expansion, or degradation. By integrating long-term multi-source datasets and cloud-based geospatial computing, this study provides a scalable and reproducible framework for assessing land cover change and vegetation stability in arid and semi-arid regions. The findings enhance understanding of dryland ecosystem dynamics under climate change and support large-scale ecological assessment in data-scarce environments.

## 1. Introduction

Land use and land cover change (LUCC) is a central topic in global environmental change research. LUCC is driven by both natural environmental factors and socioeconomic processes, and it significantly impacts ecosystem functions and environmental processes, especially in arid inland regions [1,2]. Vegetation, as a key component of terrestrial land cover, plays an essential role in maintaining ecological balance, preserving biodiversity, and regulating climate [3]. Moreover, climate change alters the spatial distribution of vegetation by influencing plant respiration, photosynthesis, and soil development processes [4,5]. In recent decades, global climate change has profoundly influenced vegetation dynamics in drylands through shifts in temperature regimes, precipitation patterns, and the increasing frequency of extreme weather events. These changes affect different vegetation types in distinct ways, underscoring the need to elucidate the underlying mechanisms of climate-driven vegetation responses [6,7]. Such understanding is critical for sustaining key ecosystem services—including soil stabilization, carbon sequestration, and forage provision—and for guiding effective regional ecological restoration efforts.

Asian drylands, located in the interior of the Eurasian continent, are characterized by water scarcity and significant topographic variations that result in complex climate processes. These regions are highly vulnerable, and vegetation exhibits heightened sensitivity to climate change [8,9]. Previous studies have provided important insights into vegetation responses in these regions. For example, grasslands, dominated by annual and perennial herbs, are highly sensitive to water-related variables such as precipitation, soil moisture, and evapotranspiration. The combined effects of climate change and overgrazing have contributed to widespread grassland degradation [10,11]. Climate change has also had significant impacts on wetland ecosystems such as Bayinbuluk Wetland, Ebinur Lake, and Bosten Lake, with intensified human activities leading to wetland shrinkage, water quality deterioration, and ecological function degradation [12,13]. Additionally, forests, regarded as important carbon sinks under climate change, exhibit significant responses to both climate variability and land use changes, with recent studies indicating large-scale cropland expansion and grassland loss in regions like Xinjiang [14–16]. These findings collectively demonstrate that vegetation dynamics in Asian drylands are highly heterogeneous and closely linked to hydroclimatic conditions.

Despite significant advances in understanding vegetation-climate interactions, most studies have relied primarily on long-term vegetation indices to investigate climate-vegetation relationships, often focusing on the greenness trends of individual vegetation types [17–19]. However, such studies have generally overlooked land cover transitions and their responses to climate change. Recent NDVI-based studies have further examined the combined effects of climate variability and anthropogenic activities on vegetation dynamics [20,21]. However, these studies primarily focus on vegetation greenness and do not directly quantify land cover transition pathways or assess vegetation stability in terms of long-term persistence versus instability. Changes in vegetation greenness do not necessarily indicate structural ecosystem change, as vegetation indices primarily reflect canopy greenness and phenological changes rather than explicit land cover conversions [22].

The development of cloud computing geospatial analysis platforms, particularly Google Earth Engine (GEE), has provided new technological capabilities for land cover change research [23,24]. By integrating global land cover products such as MODIS MCD12Q1, previous studies have quantitatively analyzed land cover changes and transition processes in various regions [13,25–27]. However, such studies still have two notable limitations. First, most studies focus on national or sub-regional scales, making it difficult to fully reveal the spatial heterogeneity and regional contrasts of Asian drylands as an integrated system. Second, although methods such as transition matrices and transition probabilities have been used to describe changes between land cover types, there is still a lack of quantitative assessments focused on vegetation stability or persistence. This makes it difficult to effectively distinguish between long-term stable regions and those undergoing highly dynamic or unstable transitions [28].

Building on these research gaps, this study integrates long-term MODIS MCD12Q1 land cover data, climate reanalysis data, and the computational advantages of Google Earth Engine to systematically assess land cover dynamics across Asian drylands from 2001 to 2022. Specifically, this study aims to: (1) quantify the spatiotemporal patterns of major land cover types; (2) identify land cover transition pathways and major transformation processes through transition probability and transition matrix analyses; (3) evaluate vegetation dynamics and stability by delineating vegetation change zones and quantifying their spatiotemporal patterns using the TDI and SDI; (4) examine the relationships between climate change and different vegetation change zones. By systematically linking land cover transitions, vegetation stability indicators, and climate drivers at a continental scale, this study provides a scalable and reproducible framework for long-term monitoring of ecosystem stability in dryland regions under climate change.

## 2. Materials and methods

### 2.1. Study area

**Fig 1:**
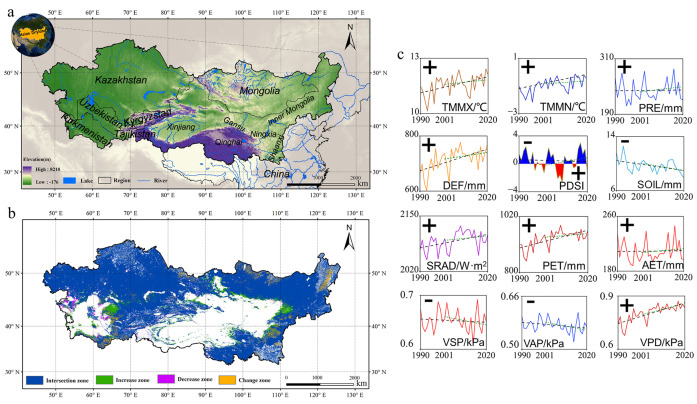
Study area:(a) Asian drylands, which mainly includes Kazakhstan (KAZ), Turkmenistan (TKM), Kyrgyzstan (KGZ), Tajikistan (TJK), Uzbekistan (UZB), Mongolia and the western provinces of China (Xinjiang, Gansu, Qinghai, Ningxia, Shaanxi, and Inner Mongolia). **(b)** Spatial distribution of vegetation change zones in Asian drylands, including intersection, increase, decrease, and change zones. (NO.: GS (2016)2966). **(c)** Overview of study area and the study’s targeted climate factor changes, i.e., TMMX, TMMN, PRE, DEF, PDSI, SOIL, SRAD, PET, AET, VSP, VAP, and VPD, from 1990 to 2020.

The Asian drylands, bounded by longitudes 46°-127°E and latitudes 31°-56°N, span the hinterland of the Eurasian continent and cover more than 10 million square kilometers. With an average elevation of 1269 meters above sea level, this region is highly undulating, ranging from −176 meters to 8218 meters, and includes the Pamir Plateau, the Tian Shan Mountains, the Mongolian Plateau, the Loess Plateau sector, and the Tibetan Plateau Focusing on the intersection zones of vegetation types over 22 years (excluding areas of changing succession), the proportions are 92.55% Grasslands (GRA), 4.56% Croplands (CRO), 1.12% Savannas (SAV), 0.56% Deciduous Broadleaf Forests (DBF), 0.51% Shrublands (SH), 0.33% Permanent Wetlands (WET), 0.31% Mixed Forests (MF), and 0.07% Needle-leaf Forests (NF). The arid and semi-arid climate inherent to Asian drylands is characterized by low annual precipitation, high summer temperatures, high actual evapotranspiration, and restricted soil moisture [29,30] (Fig 1).

### 2.2 Data

The MODIS MCD12Q1 land cover product, TerraClimate climate reanalysis data, digital elevation model (DEM) data, and CMIP6 climate projection datasets were selected to ensure temporal continuity, spatial consistency, and suitability for large-scale dryland analysis. The MODIS MCD12Q1 dataset provides annual global land cover classifications based on the IGBP scheme at a native spatial resolution of 500 m and has been widely used in large-scale land use and land cover change studies due to its long temporal coverage and consistent classification system [26,31]. TerraClimate is a global, monthly climate and water balance reanalysis dataset with a spatial resolution of approximately 4 km (1/24°) [32]. It provides internally consistent variables, including temperature, precipitation, soil moisture, evapotranspiration, and drought indices, and is particularly suitable for ecohydrological and vegetation–climate studies in data-scarce dryland regions. In addition, DEM data from the SRTM dataset were used to characterize topographic conditions [33], and CMIP6 climate projection data under SSP2–4.5 and SSP5–8.5 scenarios were employed to assess potential future climate trends.

To ensure spatial consistency for joint analysis, all datasets were harmonized to the native 4 km (1/24°) grid of the TerraClimate product. Specifically: (1) MODIS MCD12Q1 annual land cover maps (500 m) were upscaled using the majority (mode) aggregation method to retain the dominant land cover class within each 4 km grid cell. (2) The SRTM DEM (30 m) was resampled to 4 km using bilinear interpolation, which is appropriate for continuous elevation data, preserving topographic gradients without introducing significant artifacts. (3) CMIP6 daily temperature and precipitation data (0.25°) were not spatially resampled and were used solely for qualitative comparison of long-term climate trends and future scenarios. These data were not involved in pixel-level matching with observational datasets. For temporal aggregation of TerraClimate monthly variables, annual means were calculated for temperature-related variables (TMMX, TMMN), soil moisture (SOIL), vapor pressure variables (VAP, VSP, VPD), PDSI, SRAD, PET, AET, and DEF, while precipitation, AET, and PET were accumulated as annual totals before trend analysis. Detailed information on the data products and sources is provided in Table 1.

**Table 1:** Data product type and source.

*Product*	*Type*	*Temporal resolution*	*Spatial resolution*	*URL source*
***MCD12Q1***	Landcover (IGBP)	Yearly	500 m	https://modis.gsfc.nasa.gov/
***TerraClimate***	Actual evapotranspiration (AET)	Monthly	1/24° ~ 4 km	https://www.ecmwf.int
***TerraClimate***	Climate water deficit (DEF)	Monthly	1/24° ~ 4 km	https://www.ecmwf.int
***TerraClimate***	Reference evapotranspiration (PET)	Monthly	1/24° ~ 4 km	https://www.ecmwf.int
***TerraClimate***	Precipitation accumulation (PRE)	Monthly	1/24° ~ 4 km	https://www.ecmwf.int
***TerraClimate***	Soil moisture (SOIL)	Monthly	1/24° ~ 4 km	https://www.ecmwf.int
***TerraClimate***	Downward surface shortwave radiation (SRAD)	Monthly	1/24° ~ 4 km	https://www.ecmwf.int
***TerraClimate***	Maximum temperature (TMMX)	Monthly	1/24° ~ 4 km	https://www.ecmwf.int
***TerraClimate***	Minimum temperature (TMMN)	Monthly	1/24° ~ 4 km	https://www.ecmwf.int
***TerraClimate***	Vapor saturated pressure (VSP)	Monthly	1/24° ~ 4 km	https://www.ecmwf.int
***TerraClimate***	Vapor actual pressure (VAP)	Monthly	1/24° ~ 4 km	https://www.ecmwf.int
***TerraClimate***	Vapor pressure deficit (VPD)	Monthly	1/24° ~ 4 km	https://www.ecmwf.int
***TerraClimate***	Palmer Drought Severity Index (PDSI)	Monthly	1/24° ~ 4 km	https://www.ecmwf.int
***SRTM***	Digital Elevation Model (DEM)	—	30 m	https://www.usgs.gov/
***CMIP6***	Temperature (SSP245 and SSP585)	Daily	0.25°	https://www.nccs.nasa.gov/
***CMIP6***	Precipitation(SSP245 and SSP585)	Daily	0.25°	https://www.nccs.nasa.gov/

### 2.3. Methods

Based on the Google Earth Engine (GEE) and ArcGIS platforms, this study integrates MODIS land cover data (MCD12Q1), TerraClimate climate reanalysis data, and CMIP6 datasets to construct a stepwise and systematic research framework for comprehensively analyzing land cover dynamics, vegetation type transitions, and their climatic driving mechanisms across Asian drylands during 2001–2022.

First, annual MODIS MCD12Q1 land cover data were reclassified into major vegetation and non-vegetation types, and temporal and spatial dynamic indices were applied to quantitatively characterize the rate, direction, and intensity of land cover changes. Second, land cover transition probabilities and transfer matrix methods were employed to explicitly identify conversion pathways among different vegetation types as well as between vegetation and non-vegetation classes, thereby determining the dominant land cover transition processes within the study area. Subsequently, TerraClimate reanalysis data were incorporated to analyze temperature, precipitation, and moisture-related climatic factors, and atmospheric moisture conditions closely associated with vegetation dynamics were represented through the calculation of variables such as vapor saturated pressure. Finally, a grid-based statistical approach was used to classify land cover changes into intersection, increase, decrease, and transition zones. On this basis, long-term climate trends and their attribution were systematically analyzed for different vegetation dynamic zones, enabling an integrated linkage among land cover changes, vegetation stability characteristics, and climatic driving factors. The main analytical methods and models applied in this study are described in detail in Sections 2.3.1–2.3.4.

#### 2.3.1 Land cover dynamics index.

The analysis of temporal variation in land cover was conducted using the time dynamics index (TDI), which captures the variations in land cover change rates over the study period [34,35]. The formula for calculating TDI is as follows:


$$D_{T}=\frac{\sum_{\text{1}}^{s}\Delta L_{(t)j}}{L_{(t-\text{1})i}}\times \text{100}\%$$ (1)

where ***D***_***T***_ represents the TDI of type ***i****,* ***t*** represents the year being analyzed, ***L***_***(t-1)i***_ represents the area of type ***i*** in the previous year (***t-1***), ***ΔL***_***(t)j***_ denotes the change in land cover area from type ***i*** to type ***j*** in the current year ***t***, and ***s*** represents the total number of land cover changes from type ***i*** to non-type ***i*** in that year.

To quantify the extent of land cover change in a particular area, the spatial dynamics index (SDI) is utilized, which aids in identifying areas with significant land cover change. The SDI formula is as follows:


$$D_{s}=\frac{\sum_{\text{1}}^{m}\Delta L_{j}}{L_{i}}\times \text{100}\%$$ (2)

where ***D***_***S***_ represents the spatial distribution index of type ***i***, ***L***_***i***_ denotes the initial area of type ***i***, ***ΔL***_***j***_ represents the changed land cover area from type ***i*** to ***j*** at the end of the study, and ***m*** represents the total number of land cover changes from type ***i***.

#### 2.3.2 Land cover transition probability and transition matrix.

To determine the direction of land cover change, we utilized the transition probability method, as described in [34,36], to quantify the likelihood of transition between different land cover types. This approach enables the identification of hotspot types in specific transition changes and provides an accurate representation of the transitions between different types. The formula for calculating transition probability is as follows:


$$P=\frac{P_{j}}{P_{i}}\times \text{100}\%$$ (3)

where ***P*** represents the transition probability, which represents the percentage of the area transitioning from land cover type ***i*** to type ***j***. ***P***_***i***_ represents the total area of land cover type ***i*** at the start time, and ***P***_***j***_ represents the area that changed from type ***i*** to ***j*** during the study period.

In addition, we employed the transfer matrix analysis method proposed by [34,36] to examine the spatial structure of land cover transition. This method allows for the identification of regional variations in land cover transition and is formulated as follows:


$$\begin{array}{c} T=\left(T_{ij}\right)=\text{\textbackslash{}hspace\{0.17em}\;\}\left|\begin{array}{cccccc} T_{11} & T_{12} & T_{13} & \cdots & T_{1(n-1)} & T_{1n}\\ T_{21} & T_{22} & T_{23} & \cdots & T_{2(n-1)} & T_{2n}\\ \vdots & \vdots & \vdots & \ddots & \vdots & \vdots \\ T_{n1} & T_{n2} & T_{n3} & \cdots & T_{n(n-1)} & T_{nn} \end{array}\right| \end{array}$$ (4)

where ***T*** represents the matrix that describes the transitions between different land cover types. The variable ***n*** denotes the total number of land cover types. The variables ***i*** and ***j*** represent the initial and final land cover types, respectively, at the beginning and end of the study period. ***T***_***ij***_ represents the specific land cover transition from type ***i*** to type ***j***.

#### 2.3.3 VSP calculation.

The calculation of vapor saturated pressure (VSP) [37,38] is derived from reanalysis data products of TerraClimate [32], specifically the actual vapor pressure (VAP) and vapor pressure deficit (VPD). The calculation is as follows:


VSP=VAP+VPD (5)

#### 2.3.4 Grid statistics.

**Fig 2:**
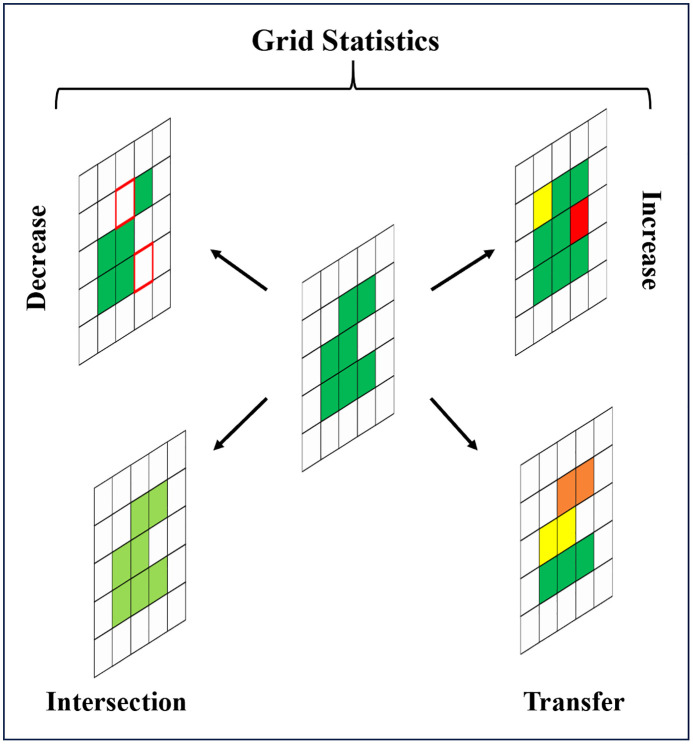
Grid statistics process roadmap.

In the processing of land use product data and the selection of climate zones, we conducted a complex grid statistics process on the GEE big data platform, including grid classification, transfer, replacement, addition, and reduction processes. Over time, increased areas are areas of increased vegetation relative to bare land; decreased areas are areas of reduced vegetation relative to bare land; transferred areas are mutual succession between vegetation types; and intersection areas are areas where no changes occur between vegetation types. The specific process is shown in the roadmap below(Fig 2).

## 3. Results

### 3.1 Spatial distribution characteristics of land cover

**Fig 3:**
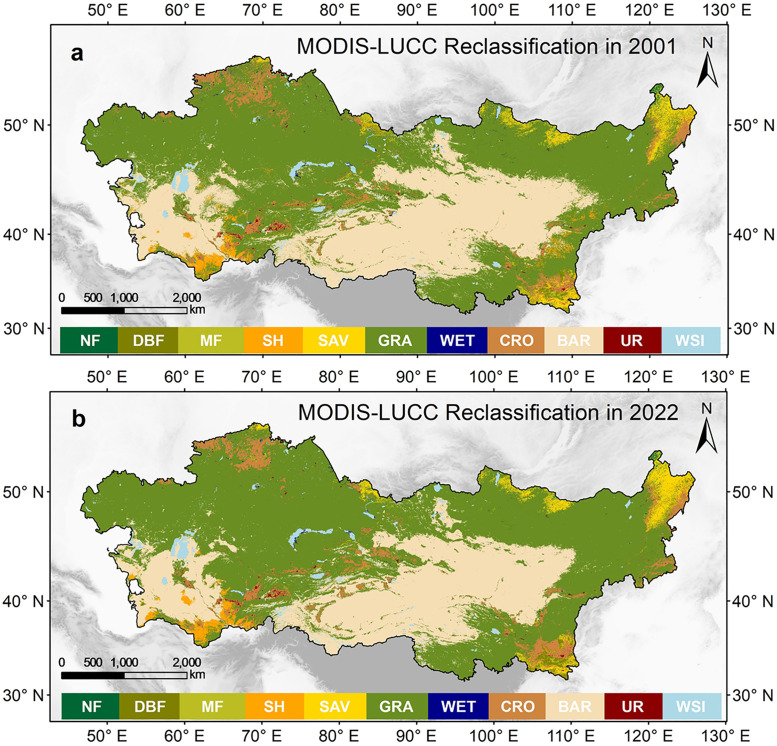
Spatial distribution of land covers in 2001 and 2022. Land cover types: **(a)** needleleaf forests, **(b)** deciduous broadleaf forests, **(c)** mixed forests, **(d)** shrublands, **(e)** savannas, **(f)** grasslands, **(g)** permanent wetlands, **(h)** croplands, **(i)** vegetation, **(j)** barren, (k) urban, **(l)** WSI (Water, Snow, and Ice). Only vegetation biomes are considered and classes in IGBP were grouped as indicated in parentheses in Fig 1b: NF = Needle-leaf Forest (1 + 3), DBF = Decidious Broadleaf Forest (4), MF = Mixed Forest (5), SH = Shrublands (6 + 7), SAV = Savannas (8 + 9), GRA = Herbaceous (10), WET = Permanent Wetlands (11), CRO = Cultivated (12 + 14), BAR = Barren (16), UR = Urban and Built-up Lands (13), WSI (Permanent Snow and Ice and Water Bodies(15 + 17)).

The distribution of land use types in 2001 and 2022 is illustrated in the figure below. Grassland and bare land dominate the study area and exhibit a patchy pattern. Bare land primarily consists of deserts, sandy areas, and desert regions within the continent, while grassland is widely distributed around these bare land areas. Forest land is mainly concentrated in mountainous regions, with different types of forest land showing latitudinal and vertical distribution patterns. Wetland is primarily found in river and lake areas and subalpine regions. Cultivated land is mainly concentrated in the oases of the central mountainous plain, as well as in the northwest, northeast, and southeast regions (Fig 3a and 3b).

In 2001, the proportions of each land use type in the dryland area of Asia were as follows: Needle-leaf Forest (NF) 0.19%, Deciduous Broadleaf Forest (DBF) 0.50%, Mixed Forest (MF) 0.44%, Shrubland (SH) 1.53%, Savannas (SAV) 2.20%, Grassland (GRA) 55.99%, Permanent Wetland (WET) 0.20%, Cropland (CRO) 4.15%, Bare land (BAR) 32.95%, Urban (UR) 0.31%, and Water and Snow/Ice (WSI) 1.53%. In 2022, the proportions were: NF 0.20%, DBF 0.86%, MF 0.35%, SH 1.26%, SAV 2.48%, GRA 58.31%, WET 0.12%, CRO 4.55%, BAR 29.85%, UR 0.32%, and WSI 1.70%. Compared to 2001, there is spatial expansion of NF, DBF, SAV, GRA, CRO, UR, and WSI in 2022, while MF, SH, WET, and BAR show spatial contraction (Fig 3a and 3b).

**Fig 4:**
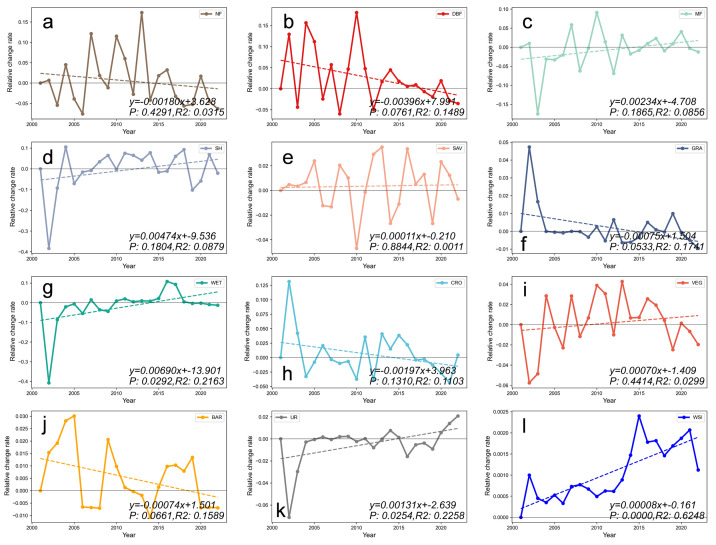
Relative changes of 12 land covers during 2001-2022. **(a)** needleleaf forests, **(b)** deciduous broadleaf forests, **(c)** mixed forests, **(d)** shrublands, **(e)** savannas, **(f)** grasslands, **(g)** permanent wetlands, **(h)** croplands, **(i)** vegetation, **(j)** barren, (k) urban, **(l)** WSI (Water, Snow, and Ice).

Comparing the relative changes in the area of 12 land use types (including all vegetation regions) from 2001 to 2022, we find that MF, SH, SAV, WET, VEG, UR, and WSI have shown an upward trend. Among these, the vegetation area and WET have the highest upward trends, with a value of 0.0069 (in terms of grid cell change). The lowest upward trend is observed in SAV, with a value of 0.00011. Conversely, NF, DBF, GRA, CRO, and BAR have shown a downward trend. In the vegetation area, DBF has the highest downward trend, with a value of 0.00396, while GRA has the lowest downward trend, with a value of 0.00075. In the non-vegetation area, BAR has a downward trend of 0.00074 (Fig 4a-4l).

These changes may be influenced by a combination of factors, including regional climate change, land policy adjustments, and increased human activities. In particular, climate warming may affect the growth of coniferous forests in high-latitude regions, while simultaneously promoting the expansion of heat-tolerant land types, such as shrubs and grasslands [39,40]. The development of wetland and aquatic vegetation types is directly influenced by the distribution of water resources. Therefore, areas rich in water resources, such as rivers, lakes, and wetlands, will facilitate the growth of wetland vegetation [41].

### 3.2 Spatio-temporal dynamic indices of land cover

**Fig 5:**
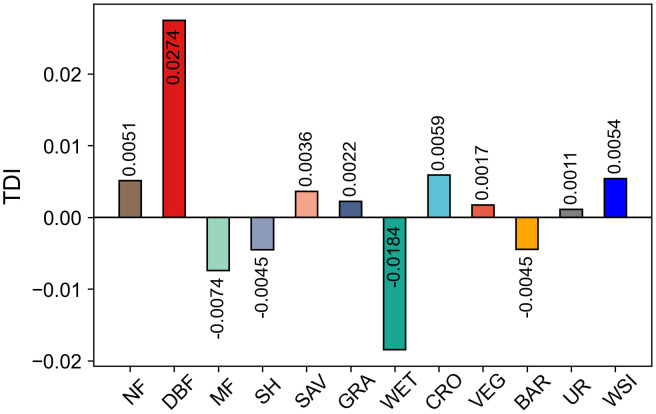
Multi-year average TDI for each land cover.

Examining the average Temporal Dynamic Index (TDI) for the period from 2001 to 2022, NF, DBF, SAV, GRA, CRO, VEG, UR, and WSI all have positive TDI values. Among these, DBF shows the highest TDI among vegetation types, whereas GRA has the lowest TDI within vegetation categories, while UR exhibits the lowest positive TDI among all land cover types. The positive TDI value of UR is lower than that of WSI. Conversely, MF, SH, WET, and BAR have negative TDI values. Among these, WET has the highest negative TDI value, while SH has the lowest negative value (Fig 5).

**Fig 6:**
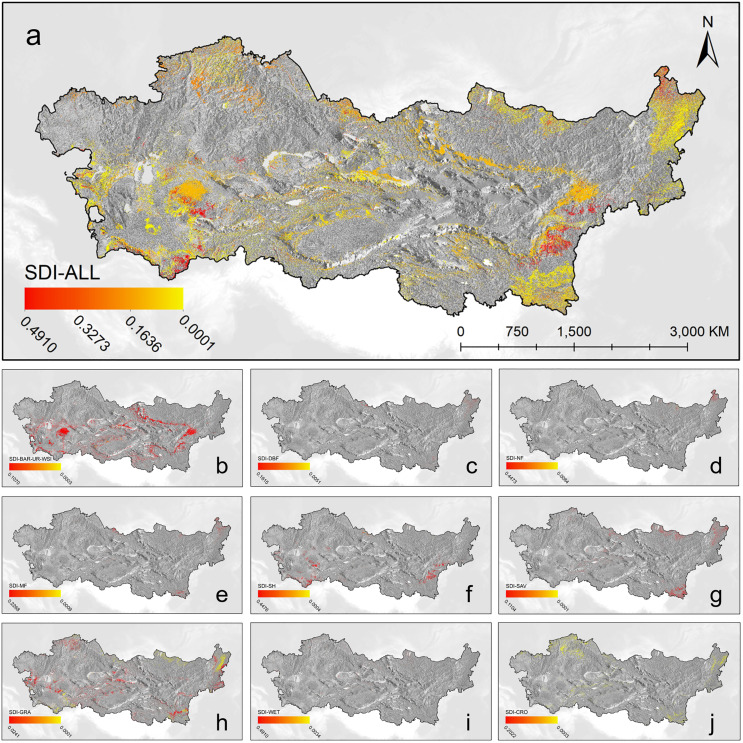
Multi-year average SDI for each land cover. Land cover types: **(a)** All of land cover (SDI ≠ 0), **(b)** BAR (barren)-UR (urban)-WSI (Water, Snow, and Ice), **(c)** deciduous broadleaf forests, **(d)** needleleaf forests, **(e)** mixed forests, **(f)** shrublands, **(g)** savannas, **(h)** grasslands, **(i)** permanent wetlands, **(j)** croplands.

Examining the average Spatial Dynamic Index (SDI) for the period from 2001 to 2022, the SDI ranges from 0.0001 to 0.4910. Regions with high SDI values are mainly distributed in the northeast, southeast, and southwest areas, primarily due to variations in NF and SH. Regions with low SDI values are distributed throughout the entire area, mainly in non-vegetation areas, with the CRO region and certain areas of GRA being relatively stable (Fig 6a-6j). The Asian drylands are susceptible to frequent natural disasters such as droughts and dust storms. These disasters can result in vegetation destruction and changes, thereby affecting the spatiotemporal dynamics of land cover [42]. For instance, prolonged droughts may lead to degradation in grassland and shrub areas, while dust storms may cause an expansion in bare land areas [43,44]. Overall, the dynamic index results based on TDI and SDI suggest that land cover changes in Asian drylands are spatially heterogeneous, with certain regions experiencing intensive transformations while others remain relatively stable over the study period.

### 3.3 Transitional changes in land cover transfer

**Fig 7:**
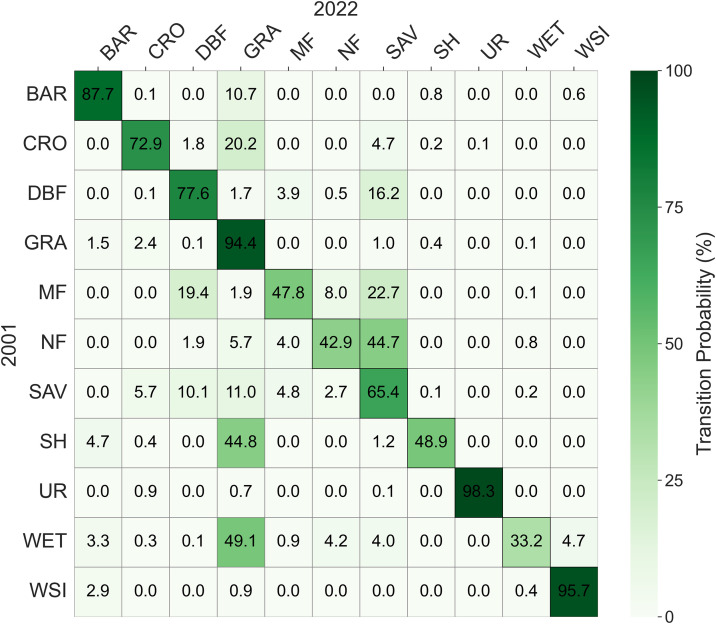
Transition probability matrix of land cover types from 2001 to 2022, highlighting dominant conversion pathways, particularly transitions from BAR to GRA and from GRA to CRO.

In terms of vegetation transition proportions (excluding the proportion that remains unchanged, as shown in the graph), NF to SAV has a high transition proportion of 44.7%, while NF to WET has a low proportion of 0.4%. DBF to SAV has a relatively high transition proportion of 16.2%. MF to SAV has a high transition proportion of 22.7%, whereas MF to GRA has a low proportion of 1.9%. SH to GRA has a high transition proportion of 44.8%. SAV to DBF and GRA have transition proportions of 10.1% and 11.0%, respectively, but SAV to NF has a low proportion of 2.7%. GRA to CRO has a transition proportion of 2.4%. WET to GRA has a high transition proportion of 49.1%, and WET to MF has a proportion of 9.0%. CRO to GRA has a transition proportion of 20.2%. BAR to GRA has a transition proportion of 10.7%, and BAR to WSI has a proportion of 6.3%. Transitions from UR and WSI to other land uses have relatively low proportions (Fig 7). These dominant transition pathways indicate that vegetation expansion and agricultural conversion represent the primary land cover change processes in Asian drylands during the study period.

**Fig 8:**
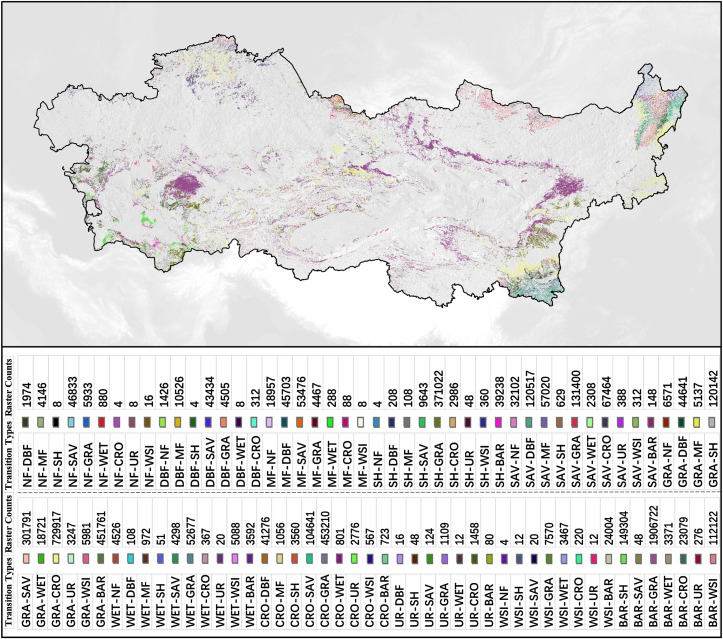
Spatiotemporal dynamics of land cover transitions across the Asian drylands from 2001 to 2022. (Note: In the table, “Transition Types” represent transitions from the left-hand type to the right-hand type, and “Raster Counts” indicate the number of pixels involved in the transition).

The specific spatial distribution and grid count (500 × 500 m) of each transition type are shown in Fig 8. The transition with the largest area is BAR to GRA (bare ground to grassland), and the vegetation type with the largest transition area is GRA to CRO (grassland to cropland). The conversion of vegetation types in arid regions may be influenced by factors such as the distribution of water resources, topographic conditions, climate types, and soil types [45,46]. For example, in mountainous areas of arid regions, coniferous forests (NF) or shrublands (SH) may be better suited for growth, while flatland areas may be more suitable for cultivating croplands (CRO) or grasslands (GRA) [41]. Additionally, the expansion of croplands (CRO) may be influenced by irrigated agriculture, and natural environmental conditions such as soil types and topography also affect the distribution and conversion of vegetation types in arid regions, thereby influencing the conversion ratios of other types [47]. Overall, the transition analysis reveals that land cover changes in Asian drylands are dominated by a limited number of major conversion pathways, with clear spatial heterogeneity in both transition direction and intensity.

### 3.4 Climate attribution in natural vegetation dynamic zones

In this study, climate trends are described in terms of their long-term magnitude and direction across different vegetation stability zones. Unless otherwise stated, the reported trends refer to descriptive patterns rather than formal statistical significance testing.

#### 3.4.1 Climatic trend analysis of natural vegetation dynamic intersection zones.

**Fig 9:**
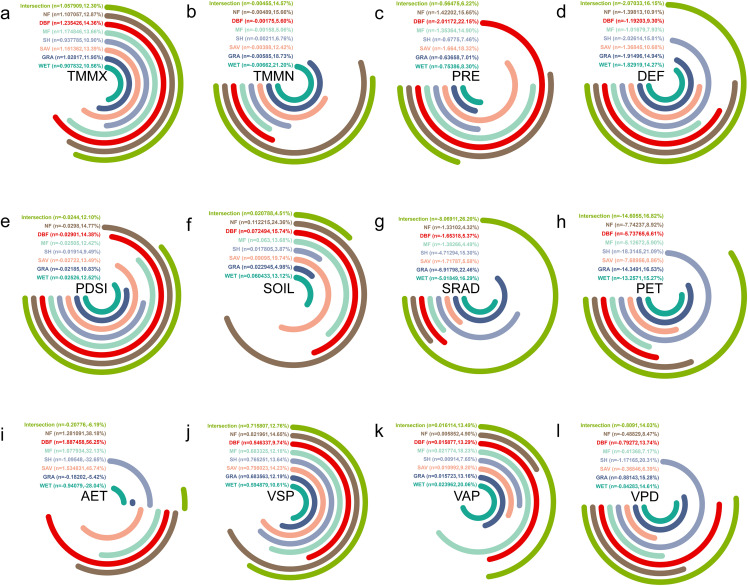
Climate factor trends for the intersection zone of the same vegetation types from 2001 to 2020 in Asian drylands. **(a)**, **(b)**, **(c)**, **(d)**, **(e)**, **(f)**, **(g)**, **(h)**, **(i)**, **(j)**, **(k)**, and (l) show trends for TMMX, TMMN, PRE, DEF, PDSI, SOIL, SRAD, PET, AET, VSP, VAP, and VPD, respectively. Note: n is climate trend, percent (%) is ranked size comparison. “Clockwise” indicates a positive trend (i.e., n > 0), and “Counterclockwise” indicates a negative trend (i.e., n < 0).

We calculated 12 climate trends (TMMX, TMMN, PRE, DEF, PDSI, SOIL, SRAD, PET, AET, VSP, VAP, and VPD) for intersection zones of natural vegetation types. Over the past 22 years, TMMX, SOIL, VSP, and VAP have all shown an upward trend across all vegetation types. Among these, DBF has the highest increasing trend in TMMX at 1.24 ℃/year, while WET has the lowest at 0.91 ℃/year. NF has the highest increasing trend in SOIL at 0.11 mm/year, while SH has the lowest at 0.02 mm/year. NF has the highest increasing trend in VSP at 0.82 kPa/year, while WET has the lowest at 0.59 kPa/year. WET has the highest increasing trend in VAP at 0.02 kPa/year, while NF has the lowest at 0.006 kPa/year (Fig 9).

Conversely, over the past 22 years, TMMN, PRE, DEF, PDSI, SRAD, PET, and VPD have shown a decreasing trend for all vegetation types. Among these, WET has the highest decreasing trend in TMMN at −0.007 ℃/year, while MF has the lowest at −0.002 ℃/year. DBF has the highest decreasing trend in PRE at −2.01 mm/year, while GRA has the lowest at −0.64 mm/year. SH has the highest decreasing trend in DEF at −2.03 mm/year, while MF has the lowest at −1.02 mm/year. NF has the highest decreasing trend in PDSI at −0.030/year, while SH has the lowest at −1.019/year. GRA has the highest decreasing trend in SRAD at −6.92 W·m²/year, while NF has the lowest at −1.33 W·m²/year. SH has the highest decreasing trend in PET at −18.31 mm/year, while MF has the lowest at −5.13 mm/year. SH has the highest decreasing trend in VPD at −1.17 kPa/year, while MF has the lowest at −0.41 kPa/year. Additionally, over the past 22 years, only AET has shown both increasing and decreasing trends. Among the increasing trends, DBF has the highest at 1.89 mm/year, while MF has the lowest at 1.08 mm/year. Among the decreasing trends, SH has the highest at −1.10 mm/year, while GRA has the lowest at −0.18 mm/year (Fig 9).

Global warming, greenhouse gas emissions, and human activities have led to an increase in Earth’s surface temperature, affecting temperature indicators and potentially causing changes in precipitation patterns, influencing surface water circulation, and evaporation processes [48,49]. Natural disasters such as droughts, floods, and extreme weather events may result in short-term changes in climate indicators, such as decreased precipitation and increased net radiation. Ecosystems have feedback effects on climate change; for example, changes in vegetation may affect surface temperature and evaporation processes [34]. Additionally, actual evapotranspiration shows trends of both increase and decrease, influenced by a combination of factors including climate, soil, vegetation, and human activities. These trends provide an in-depth understanding of the health and stability of respective ecosystems, offering important insights for future ecological conservation and climate change adaptation.

#### 3.4.2 Climatic trend analysis of natural vegetation dynamic increase zones.

**Fig 10:**
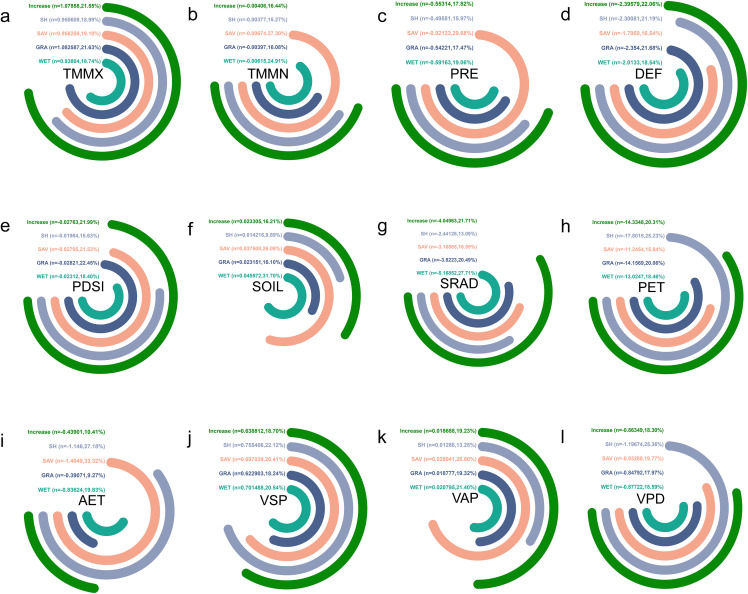
Climate factor trends for the increase zone of the same vegetation types from 2001 to 2020 in Asian drylands. **(a)**, **(b)**, **(c)**, **(d)**, **(e)**, **(f)**, **(g)**, **(h)**, **(i)**, **(j)**, **(k)**, and (l) show trends for TMMX, TMMN, PRE, DEF, PDSI, SOIL, SRAD, PET, AET, VSP, VAP, and VPD, respectively. Note: n is climate trend, percent (%) is ranked size comparison. “Clockwise” indicates a positive trend (i.e., n > 0), and “Counterclockwise” indicates a negative trend (i.e., n < 0).

The main vegetation types in the increased area are SH, SAV, GRA, and WET (We have ignored grid cells with single-digit values, the same applies below). Over the past 22 years, we found that TMMX, SOIL, VSP, and VAP have shown an increasing trend. Among these, GRA has the highest increasing trend in TMMX at 1.08 ℃/year, while WET has the lowest at 0.93 ℃/year. SAV has the highest increasing trend in SOIL at 0.04 mm/year, while SH has the lowest at 0.01 mm/year. SH has the highest increasing trend in VSP at 0.76 kPa/year, while GRA has the lowest at 0.62 kPa/year. SAV has the highest increasing trend in VAP at 0.03 kPa/year, while SH has the lowest at 0.01 kPa/year (Fig 10). The greatest increase in maximum temperatures is observed in grassland areas, likely related to climate warming and changes in grassland ecosystems [50]. Arid regions in Asia are already experiencing dry conditions, with inherently low precipitation, resulting in relatively low humidity or precipitation levels [51].

Furthermore, over the past 22 years, TMMN, PRE, DEF, PDSI, SRAD, PET, AET, and VPD have shown a decreasing trend for all vegetation types. Among these, SAV has the highest decreasing trend in TMMN at −0.007 ℃/year, while SH has the lowest at −0.004 ℃/year. SAV has the highest decreasing trend in PRE at −0.92 mm/year, while SH has the lowest at −0.50 mm/year. GRA has the highest decreasing trend in DEF at −2.35 mm/year, while SAV has the lowest at −1.80 mm/year. GRA has the highest decreasing trend in PDSI at −0.03/year, while SH has the lowest at −0.02/year. WET has the highest decreasing trend in SRAD at −5.17 W·m²/year, while SH has the lowest at −2.44 W·m²/year. SH has the highest decreasing trend in PET at −17.80 mm/year, while SAV has the lowest at −11.25 mm/year. SAV has the highest decreasing trend in AET at −1.40 mm/year, while GRA has the lowest at −0.39 mm/year. SH has the highest decreasing trend in VPD at −1.20 kPa/year, while GRA has the lowest at −0.85 kPa/year (Fig 10). The rapid loss of heat from the surface of arid SAV, leading to a pronounced decrease in nighttime temperatures and resulting in a declining trend in TMMN, is similar to the findings of Guo et al. regarding African coefficient grasslands [51]. These trends reflect the climate characteristic changes in the region over the past two decades, which may have significant implications for ecosystems and society, warranting further research and attention.

#### 3.4.3 Climatic trend analysis of natural vegetation dynamic decrease zones.

**Fig 11:**
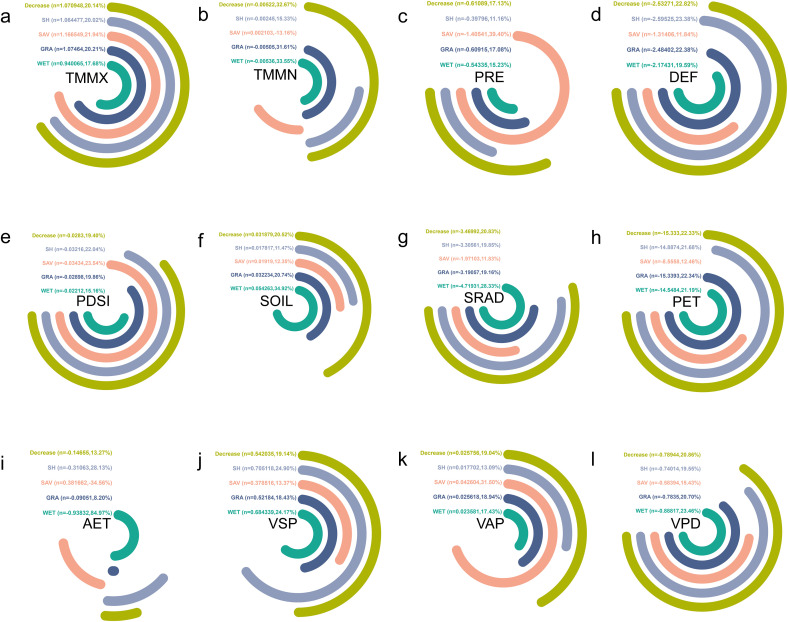
Climate factor trends for the decrease zone of the same vegetation types from 2001 to 2020 in Asian drylands. **(a)**, **(b)**, **(c)**, **(d)**, **(e)**, **(f)**, **(g)**, **(h)**, **(i)**, **(j)**, **(k)**, and (l) show trends for TMMX, TMMN, PRE, DEF, PDSI, SOIL, SRAD, PET, AET, VSP, VAP, and VPD, respectively. Note: n is climate trend, percent (%) is ranked size comparison. “Clockwise” indicates a positive trend (i.e., n > 0), and “Counterclockwise” indicates a negative trend (i.e., n < 0).

The main vegetation types in the decreased area are the same as in the increased area, namely SH, SAV, GRA, and WET. Over the past 22 years, we found that TMMX, SOIL, VSP, and VAP have shown an increasing trend. Among these, SAV has the highest increasing trend in TMMX at 1.17 ℃/year, while WET has the lowest at 0.94 ℃/year. WET has the highest increasing trend in SOIL at 0.05 mm/year, while SH has the lowest at 0.02 mm/year. SH has the highest increasing trend in VSP at 0.71 kPa/year, while SAV has the lowest at 0.38 kPa/year. SAV has the highest increasing trend in VAP at 0.04 kPa/year, while SH has the lowest at 0.02 kPa/year (Fig 11).

Furthermore, over the past 22 years, PRE, DEF, PDSI, SRAD, PET, and VPD have shown a decreasing trend for all vegetation types. SAV has the highest decreasing trend in PRE at −1.41 mm/year, while SH has the lowest at −0.40 mm/year. SH has the highest decreasing trend in DEF at −2.60 mm/year, while SAV has the lowest at −1.31 mm/year. SAV has the highest decreasing trend in PDSI at −0.03/year, while SH has the lowest at −0.02/year. WET has the highest decreasing trend in SRAD at −4.72 W·m²/year, while SAV has the lowest at −1.97 W·m²/year. GRA has the highest decreasing trend in PET at −15.34 mm/year, while SAV has the lowest at −8.56 mm/year. WET has the highest decreasing trend in VPD at −0.89 kPa/year, while SAV has the lowest at −0.58 kPa/year. Additionally, over the past 22 years, only TMMN and AET show both increasing and decreasing trends. The increasing trends in TMMN and AET are both observed in SAV (TMMN: 0.002 ℃/year and AET: 0.382 mm/year). Among the decreasing trends in TMMN, WET has the highest at −0.005 ℃/year, while SH has the lowest at −0.002 ℃/year. Among the decreasing trends in AET, SH has the highest at −0.94 mm/year, while GRA has the lowest at −0.09 mm/year (Fig 11).

Over the past two decades, ecological regions dominated by native vegetation such as SH, SAV, GRA, and WET have exhibited distinct climate interaction characteristics. Specifically, the SH system can create a local temperature and humidity climate environment through canopy transpiration [52]; GRA and WET influence soil moisture content and surface evaporation rates through root zone infiltration and water surface evaporation processes [53]. The spatiotemporal changes in surface cover may trigger alterations in regional climate variables [54]. Temperature, soil moisture content, saturation vapor pressure, and vapor pressure deficit are on the rise, while precipitation, precipitation deficit, aridity index, solar radiation, potential evapotranspiration, and evaporation deficit continue to decrease. These changes are likely influenced by a combination of factors, including land use transitions, ecosystem feedback effects, global climate change, and human activities.

#### 3.4.4 Climatic trend analysis of natural vegetation dynamic change zones.

**Fig 12:**
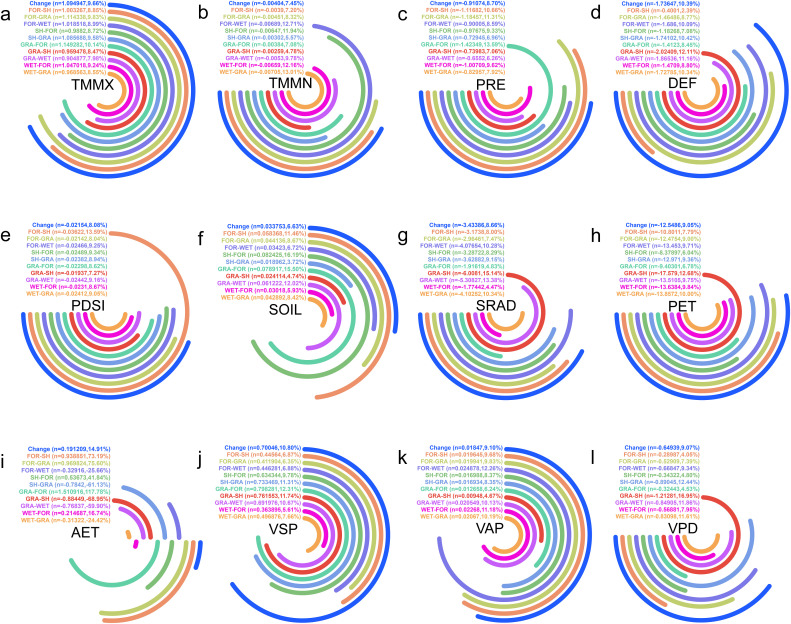
Climate factor trends for the change(transition) zone of the same vegetation types from 2001 to 2020 in Asian drylands. **(a)**, **(b)**, **(c)**, **(d)**, **(e)**, **(f)**, **(g)**, **(h)**, **(i)**, **(j)**, **(k)**, and (l) show trends for TMMX, TMMN, PRE, DEF, PDSI, SOIL, SRAD, PET, AET, VSP, VAP, and VPD, respectively. Note:n is climate trend, percent (%) is ranked size comparison. “Clockwise” indicates a positive trend (i.e., n > 0), and “Counterclockwise” indicates a negative trend (i.e., n < 0).

Due to the diverse vegetation types in the changing area, we have merged NF, DBF, MF, and SAV into a single category, FOR (Forest). The main transitions occur between FOR, SH (Shrubland), GRA (Grassland), and WET (Wetland), resulting in 10 identified transition types: FOR-SH, FOR-GRA, FOR-WET, SH-FOR, SH-GRA, GRA-FOR, GRA-SH, GRA-WET, WET-FOR, and WET-GRA. Over the past 22 years, we found that TMMX, SOIL, VSP, and VAP have shown an increasing trend for all vegetation types. Among these, GRA-FOR has the highest increasing trend in TMMX at 1.15 ℃/year, while GRA-WET has the lowest at 0.90 ℃/year. SH-FOR has the highest increasing trend in SOIL at 0.08 mm/year, while SH-GRA has the lowest at 0.02 mm/year. GRA-FOR has the highest increasing trend in VSP at 0.80 kPa/year, while WET-FOR has the lowest at 0.36 kPa/year. FOR-WET has the highest increasing trend in VAP at 0.02 kPa/year, while GRA-SH has the lowest at 0.009 kPa/year (Fig 12).

Furthermore, over the past 22 years, TMMN, PRE, DEF, PDSI, SRAD, PET, and VPD have shown a decreasing trend for all vegetation types. Among these, FOR-WET has the highest decreasing trend in TMMN at −0.007 ℃/year, while GRA-SH has the lowest at −0.003 ℃/year. GRA-FOR has the highest decreasing trend in PRE at −1.42 mm/year, while GRA-WET has the lowest at −0.66 mm/year. GRA-SH has the highest decreasing trend in DEF at −2.02 mm/year, while FOR-SH has the lowest at −0.40 mm/year. FOR-SH has the highest decreasing trend in PDSI at −0.04/year, while GRA-SH has the lowest at −0.02/year. GRA-SH has the highest decreasing trend in SRAD at −6.01 W·m²/year, while WET-FOR has the lowest at −1.77 W·m²/year. GRA-SH has the highest decreasing trend in PET at −17.58 mm/year, while SH-FOR has the lowest at −8.38 mm/year. GRA-SH has the highest decreasing trend in VPD at −1.21 kPa/year, while FOR-SH has the lowest at −0.29 kPa/year. Additionally, over the past 22 years, only AET shows both increasing and decreasing trends. Among the increasing trends, GRA-FOR has the highest at 1.51 mm/year, while WET-FOR has the lowest at 0.21 mm/year. Among the decreasing trends, GRA-SH has the highest at −0.88 mm/year, while WET-GRA has the lowest at −0.31 mm/year (Fig 12).

Overall, the results indicate that climate conditions associated with different vegetation stability zones exhibit clearly differentiated long-term trends, underscoring the heterogeneous climate–vegetation relationships across Asian drylands.

In general, changes in atmospheric circulation patterns have resulted in alterations in precipitation distribution and frequency, thereby affecting the trends of climate elements such as precipitation amount and drought indices [55]. These changes may exacerbate the occurrence and severity of drought in Asian drylands, exerting significant impacts on local agriculture, ecosystems, and socio-economic systems [56,57].

### 3.5. Future climate change trends in different regions and their potential impacts on vegetation

Building on the observed relationships between vegetation dynamics and historical climate trends, this section further examines future climate change under CMIP6 scenarios and its potential implications for vegetation. This study analyzes future trends in precipitation and temperature in the research area under the SSP2–4.5 and SSP5–8.5 scenarios using data from four CMIP6 models. The annual average temperature across all four regions shows a significant increasing trend, with the rate of temperature rise under the SSP5–8.5 scenario being more pronounced compared to the SSP2–4.5 scenario. Precipitation also generally shows an upward trend, but with greater variability. However, more intense warming could counteract the benefits of increased precipitation by enhancing evapotranspiration, resulting in a decline in actual water availability, which could lead to complex and dynamic potential impacts on vegetation [58,59].

**Fig 13:**
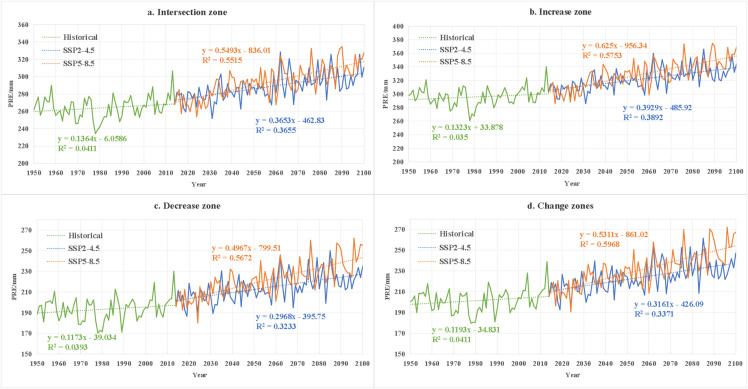
Temporal variation of precipitation in the study area during the historical period (1950-2014) and future period (2015-2100), and the average precipitation and trends under different future scenarios.

**Fig 14:**
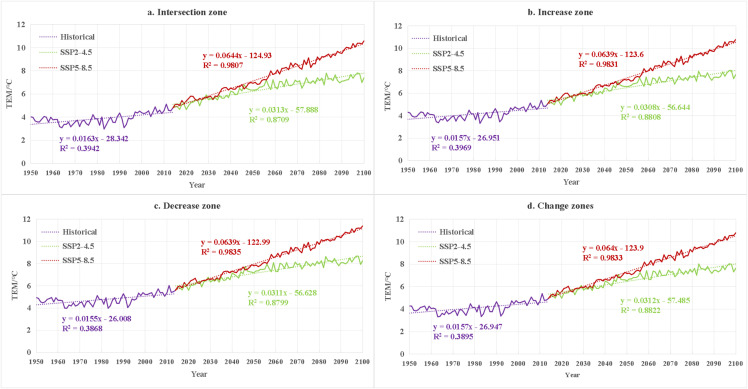
Temporal variation of average temperature in the study area during the historical period (1950-2014) and future period (2015-2100), and the average temperature and trends under different future scenarios.

To better illustrate regional contrasts, future climate trends and their potential vegetation impacts are further examined across the four vegetation change zones. From a regional perspective, in the intersection region (Figs 13a and 14a), the largest increase in precipitation occurs under the SSP5–8.5 scenario, with a rate of 0.5175 mm/year, while the temperature rise reaches 0.0312°C/year. This could intensify evaporation and undermine the ecological benefits of increased precipitation [59]. Although this region has historically been dominated by GRA and BAR, increased future precipitation could alleviate some of the drought pressure. However, the higher temperatures may encourage the expansion of drought-tolerant vegetation such as SAV, threatening the stability of NF and MF [60]. Under the SSP2–4.5 scenario, the rate of precipitation increase is slower (+0.3613 mm/year) and the temperature rise is moderate (0.0163°C/year), which would support the dynamic stability of existing vegetation. In the increase region (Figs 13b and 14b), there is a clear trade-off between the benefits of increased precipitation and heat stress. Under the SSP5–8.5 scenario, the increase in precipitation is substantial, potentially leading to vegetation expansion, but extreme warming could limit the expansion of GRA and SH due to water stress [16,61]. In the SSP2–4.5 scenario, the significant increase in precipitation compared to other regions and the moderate temperature rise could support the continued expansion of vegetation cover [62].

In the decrease region (Figs 13c and 14c), under the SSP5–8.5 scenario, the increase in precipitation is smaller compared to other regions, and extreme warming could lead to water imbalance, accelerating the GRA and their conversion into BAR. Under the SSP2–4.5 scenario, the slower rate of precipitation increase and the continuous rise in temperature could lead to soil drought, generating a negative feedback loop with the historical BAR-GRA dominant transition matrix [63]. In the change region (Fig 13d and 14d), under the SSP5–8.5 scenario, precipitation increases more than in the Decrease region, but persistent warming could disturb the balance of competition between FOR and GRA. For instance, higher temperatures may promote the invasion of GRA into forested areas, and precipitation fluctuations could accelerate vegetation reorganization, threatening the stability of ecosystem services [64]. Under the SSP2–4.5 scenario, moderate increases in precipitation and gradual warming may support a slow adjustment of vegetation, although there may also be bi-directional shifts in vegetation types at transitional zones.

In summary, the future climate warming and the variability in precipitation present complex and multifaceted effects on vegetation in arid regions. It is recommended that differentiated strategies be implemented: under the SSP5–8.5 scenario, priority should be given to the restoration of heat-tolerant vegetation and efficient water resource management, while under the SSP2–4.5 scenario, vegetation cover management should be optimized based on precipitation, with a focus on stabilizing the change region to enhance regional ecological resilience.

## 4. Discussion

### 4.1 Synthesis of key findings and comparison with previous studies

This study integrates MODIS remote sensing data, climate reanalysis data, and the Google Earth Engine (GEE) platform to conduct a large-scale, systematic assessment of land cover change and vegetation dynamics across Asian drylands from 2001 to 2022. The results indicate that vegetation greening has been the predominant trend across most Asian dryland regions over the past two decades, particularly in grassland- and shrubland-dominated ecosystems. In contrast, localized vegetation browning was observed in parts of West and Central Asia, where persistent drought conditions and increasing temperatures have intensified water stress. Overall, vegetation responses to climate change across Asian drylands exhibit significant spatial heterogeneity.

The climate attribution analysis suggests that precipitation variability is the primary driver of vegetation dynamics across most arid and semi-arid regions, whereas temperature plays a secondary role, although its effect varies regionally. Other studies have further indicated that vegetation changes in drylands are not only constrained by precipitation and soil moisture availability but may also increasingly be influenced by rising atmospheric water demand. Furthermore, under a warming climate, the increase in vapor pressure deficit (VPD) may amplify the effects of drought and inhibit vegetation growth [65,66].

The vegetation greening observed in this study aligns with several global-scale studies that report similar trends in dryland and semi-arid regions [67,68]. These studies suggest that increased precipitation and elevated atmospheric CO_2_ concentrations have jointly contributed to enhanced vegetation activity at the global scale. At the regional scale, studies focusing on Asia and Central Asia similarly report increased vegetation activity primarily driven by precipitation variability and climate fluctuations [69], which is consistent with the findings of this study. Moreover, the vegetation browning detected in certain regions of Asian drylands also concurs with growing evidence that greening trends may slow, stagnate, or even reverse under intensified climatic stress [70,71]. Recent studies suggest that browning signals can be masked by overall greening trends, and these browning signals may become more apparent when considering nonlinear vegetation responses to prolonged drought and increasing atmospheric evaporative demand [66]. These findings emphasize the nonlinear nature and potential reversibility of long-term vegetation trends in dryland ecosystems.

Compared to previous studies, this research provides a more detailed analysis of long-term vegetation changes and presents clearer spatial patterns. Furthermore, the application of the GEE platform significantly improves the efficiency of large-scale, multi-temporal satellite data processing and enhances the reproducibility and scalability of the study, effectively addressing the limitations of traditional regional-scale analyses in data processing capabilities [72,73].

### 4.2. Mechanisms of climate trends in natural vegetation cover change

**Fig 15:**
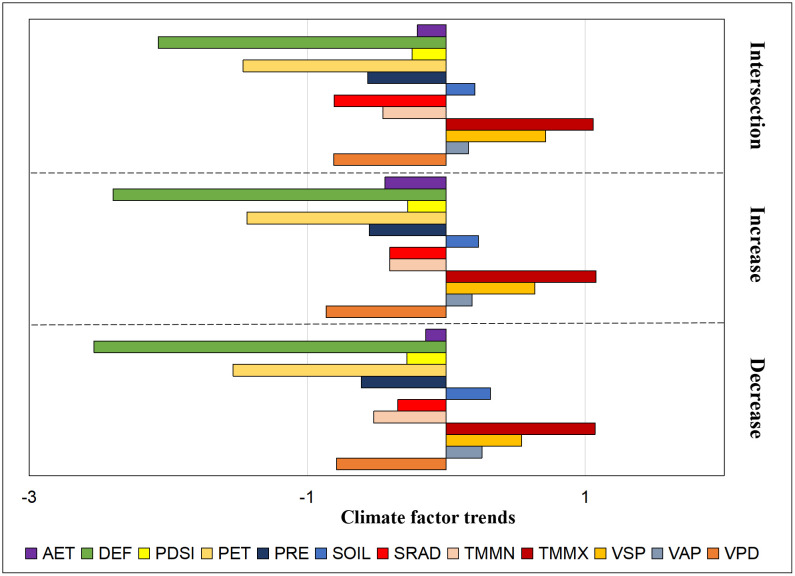
Comparison of climate change trends in vegetation intersection zones, increase zones and decrease zones.

After comparing the climate trends in the vegetation intersection zone, increase zone, and decrease zone, we found that in terms of AET, PRE, PET, TMMN, and VPD, the increase zone is developing towards a warmer and wetter direction compared to the intersection and decrease zones. Conversely, the decrease zone is developing towards a colder and drier direction compared to the intersection and increase zones. The climate trend in the intersection zone falls between the two, indicating a stable condition. However, the comparison of other climate trends is not significant due to interference from various factors, and in some cases, they even develop in opposite directions (Fig 15). For example, SOIL may be influenced by groundwater [74–76], and the levels of SRAD and TMMX can have both positive and negative effects on vegetation changes [77,78]. The changes in VAP are regulated by VSP and VPD, and similarly, the changes in VSP are regulated by VAP and VPD, resulting in unclear patterns for VAP and VPD [79]. PDSI shows weaker drought performance in the increase zone and stronger drought performance in the decrease zone, which aligns with the expected trend. However, in the intersection zone, the drought performance is the weakest, possibly due to spatial differences between the increase, decrease, and intersection zones at the regional scale.

### 4.3. Limitations and future prospects

While this study provides a systematic analysis of vegetation-climate interactions in Asian drylands using GEE and long-term remote sensing data, several limitations should be acknowledged. While the harmonization of datasets to the 4 km TerraClimate resolution enabled consistent large-scale analysis, upscaling categorical MODIS land cover via majority aggregation and bilinear interpolation of the SRTM DEM may introduce minor smoothing artifacts or overlook sub-grid heterogeneity in transitional or boundary areas, potentially affecting vegetation change zone delineation in heterogeneous dryland landscapes. In addition, the use of TerraClimate reanalysis data may introduce uncertainties, especially in arid regions with sparse ground observations, which could affect the accuracy of key variables like soil moisture (SOIL) and vapor pressure deficit (VPD). Second, despite using the standardized MODIS MCD12Q1 classification system, the lack of ground validation for land cover transitions may limit the reliability of fine-scale vegetation change detection. Third, while the study focuses on climatic drivers such as maximum temperature (TMMX), precipitation (PRE), and the Palmer Drought Severity Index (PDSI), it does not consider socio-economic factors like cropland irrigation and grazing intensity, which could influence land cover patterns in human-dominated ecosystems. Finally, future projections based on CMIP6 scenarios primarily emphasize climate-vegetation relationships but do not account for uncertainties inherent in these scenarios, which may affect the reliability of predicted vegetation trajectories.

Building on these limitations, future research could explore several avenues. First, integrating higher-resolution meteorological and ground observation data, especially in data-scarce dryland regions, would help reduce uncertainties in key climate variables like soil moisture and VPD. Second, incorporating socio-economic factors such as irrigation, grazing, and land use changes alongside climatic variables would offer a more comprehensive understanding of how human activities and climate jointly influence vegetation dynamics. Furthermore, applying region-specific climate models with higher resolution would improve projections of vegetation responses, enhancing both predictive accuracy and management strategies. Finally, focusing on ecological restoration and adaptive management, particularly in climate-stressed areas, will be crucial for ensuring ecosystem resilience, with an emphasis on drought-resistant vegetation recovery and efficient water management.

## 5. Conclusions

This study systematically analyzed land cover dynamics, vegetation type transitions, and their climatic drivers across Asian drylands from 2001 to 2022 by integrating long-term MODIS MCD12Q1 land cover data, TerraClimate climate reanalysis datasets, and the Google Earth Engine (GEE) platform. By combining land cover dynamics analysis, transition pathway identification, and climate attribution within a unified framework, this study provides a continental-scale characterization of vegetation stability and transformation processes in dryland ecosystems.

The results show that Asian drylands have experienced pronounced land cover changes over the past two decades, characterized by overall expansions of GRA, SAV, CRO, and WSI, alongside contractions of SH, MF, WET, and BAR. Land cover transition analysis further identifies dominant conversion pathways, particularly transitions from BAR to GRA and from GRA to CRO, reflecting the combined influences of climate variability and land use processes. Climate attribution analyses indicate that vegetation dynamics across different stability zones exhibit distinct responses to long-term climate trends. Increasing TMMX, SOIL, and vapor-related variables (e.g., VSP and VAP), together with declining PRE, PDSI, and SRAD, play differentiated roles in shaping vegetation persistence, expansion, or degradation across the study region. These findings highlight the importance of jointly considering vegetation transition pathways and stability characteristics when assessing dryland ecosystem responses to climate change. By leveraging cloud-based geospatial computing and multi-source long-term datasets, this study establishes a scalable and reproducible framework for monitoring land cover change and vegetation stability in arid and semi-arid regions, providing methodological support and scientific evidence for large-scale ecological assessment and adaptive land management in data-scarce environments.

**Nomenclature**

**Table:** 

*Abbreviation*	*Meaning*	*Abbreviation*	*Meaning*
AET	Actual EvapoTranspiration	SAV	Savannas
BAR	Barren	SDI	Spatial Dynamic Index
CRO	Croplands	SH	Shrublands
DBF	Deciduous Broadleaf Forest	SOIL	Soil moisture
DEF	Climate water deficit	SRAD	Downward surface shortwave radiation
DEM	Digital Elevation Model	TDI	Temporal Dynamic Index
GEE	Google Earth Engine	TMMX	Maximum temperature
GRA	Grasslands	TMMN	Minimum temperature
IGBP	International Geosphere-Biosphere Programme	UR	Urban
LUCC	Land Use and Land Cover Change	VAP	Vapor actual pressure
MF	Mixed Forest	VPD	Vapor pressure deficit
MODIS	Moderate-resolution Imaging Spectroradiometer	VSP	Vapor saturated pressure
NF	Needle-leaf Forest	WET	Permanent Wetlands
PDSI	Palmer Drought Severity Index	WSI	Water, Snow, and Ice
PET	Potential EvapoTranspiration	VEG	All vegetation types
PRE	Precipitation		

## Supporting information

S1 Dataset Geospatial data for land cover distribution maps (Fig 3). (ZIP)

S1 Table Relative change data for land cover types (Fig 4). (XLSX)

S2 Table Temporal dynamic index dataset (Fig 5). (XLSX)

S1 Fig Spatial dynamic index raster (Fig 6). (ZIP)

S3 Table Land cover transition probability matrix (Fig 7). (XLSX)

S4 Table Spatial data for major land cover transitions (Fig 8). (XLSX)

S2 Dataset Climate trend data for vegetation dynamic zones (Figs 9–12). (ZIP)

S5 Table Comparative metrics for vegetation zone climate trends (Fig 13). (XLSX)

S3 Dataset Future climate projection data (Figs 14–15). (ZIP)
