# Supplementary figures and images for: Efficient large-scale land cover change detection using Google Earth Engine: Climate-driven vegetation dynamics in Asian drylands (2001–2022)
Source: PLoS One. 2026 Apr 1;21(4):e0344835. doi: 10.1371/journal.pone.0344835 (PMC13042746; doi:10.1371/journal.pone.0344835)

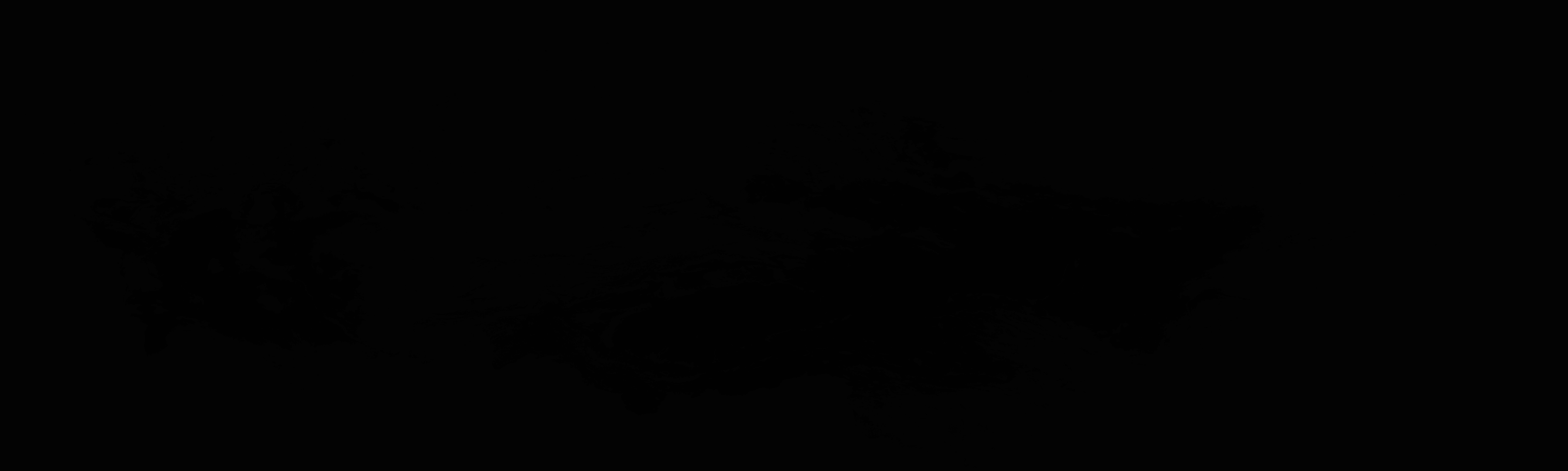

Supplement: S1 Dataset — (ZIP) [file pone.0344835.s001.zip › LUCC2022/BAR.tif]

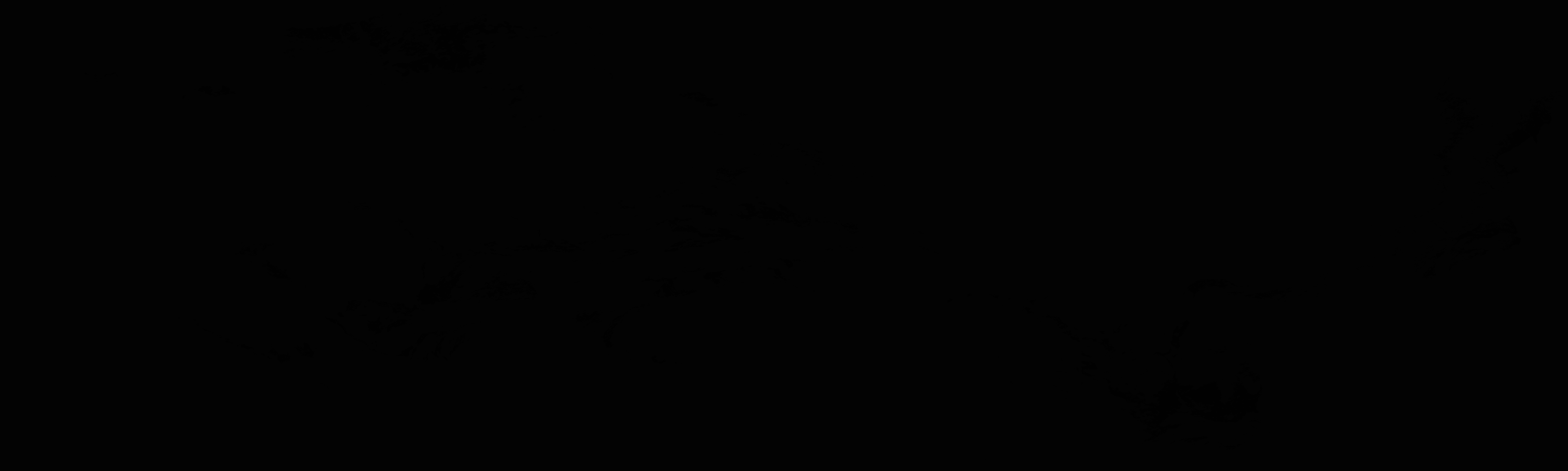

Supplement: S1 Dataset — (ZIP) [file pone.0344835.s001.zip › LUCC2022/CRO.tif]

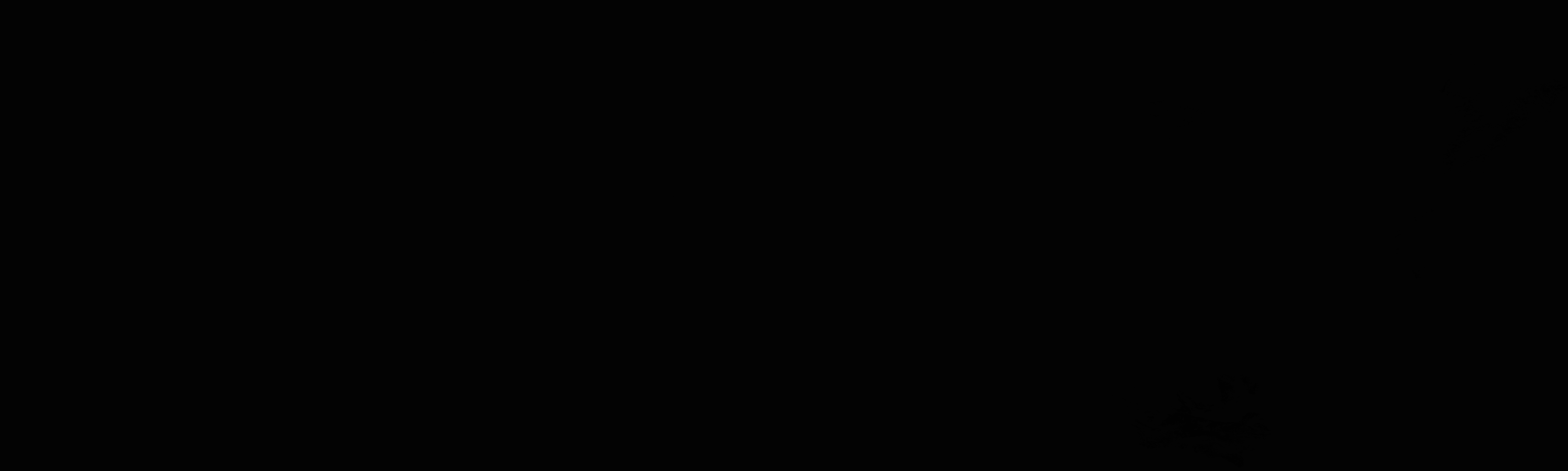

Supplement: S1 Dataset — (ZIP) [file pone.0344835.s001.zip › LUCC2022/DBF.tif]

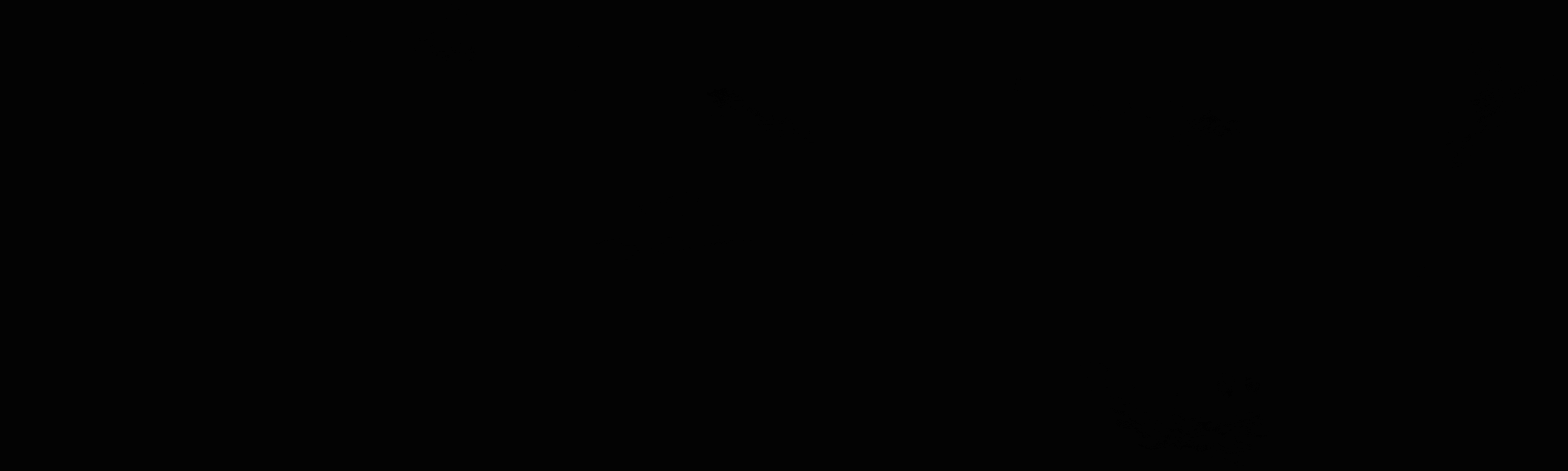

Supplement: S1 Dataset — (ZIP) [file pone.0344835.s001.zip › LUCC2022/MF.tif]

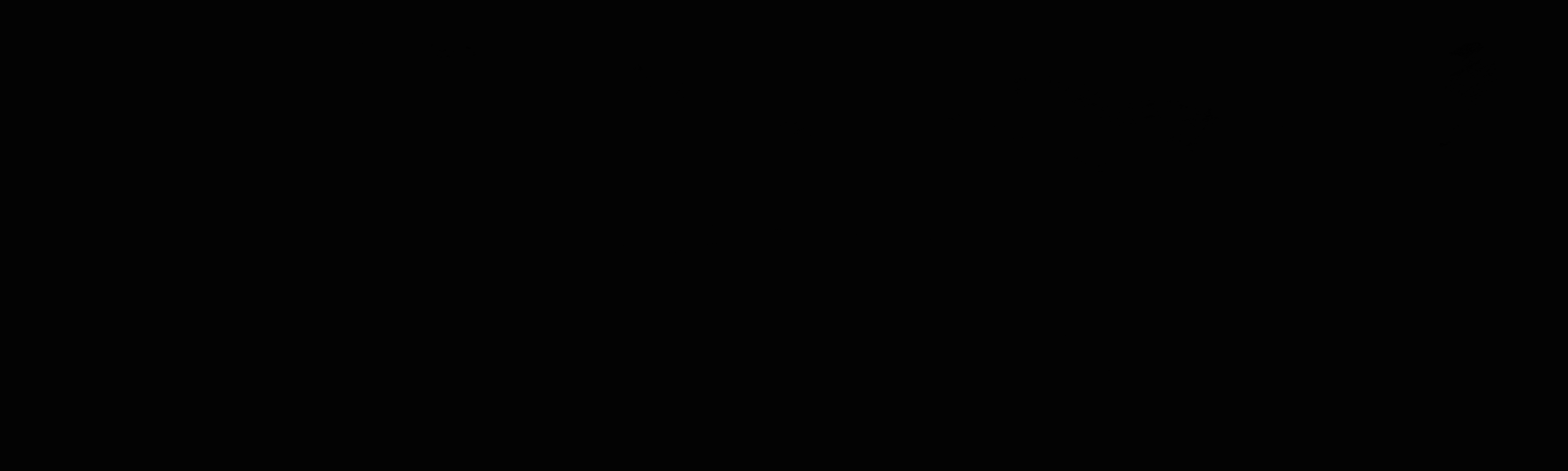

Supplement: S1 Dataset — (ZIP) [file pone.0344835.s001.zip › LUCC2022/NF.tif]

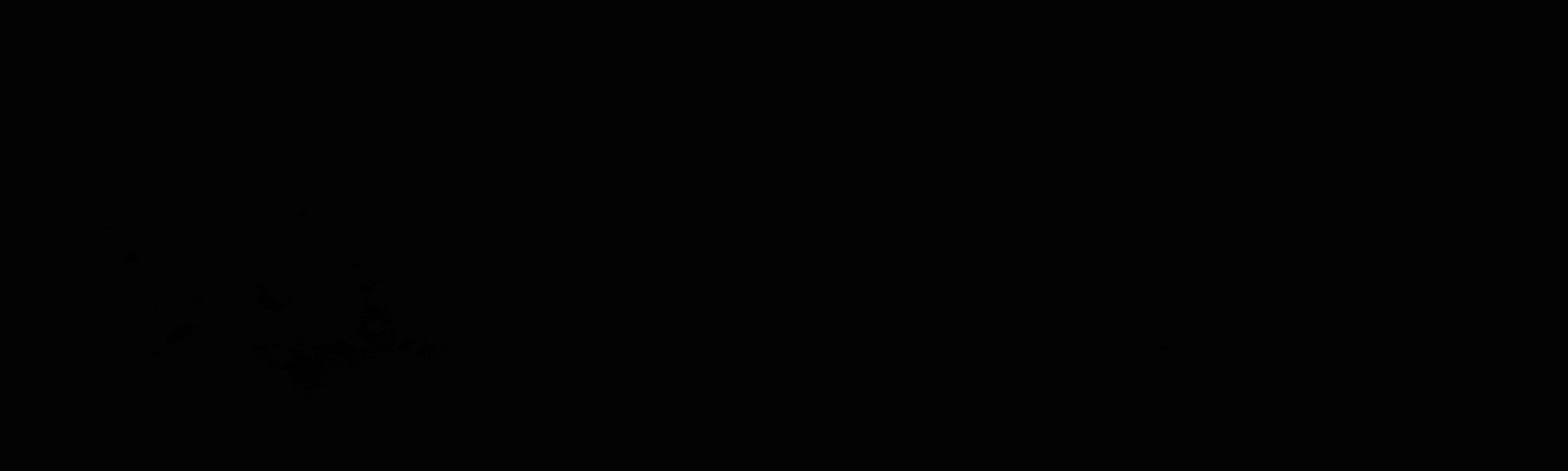

Supplement: S1 Dataset — (ZIP) [file pone.0344835.s001.zip › LUCC2022/SH.tif]

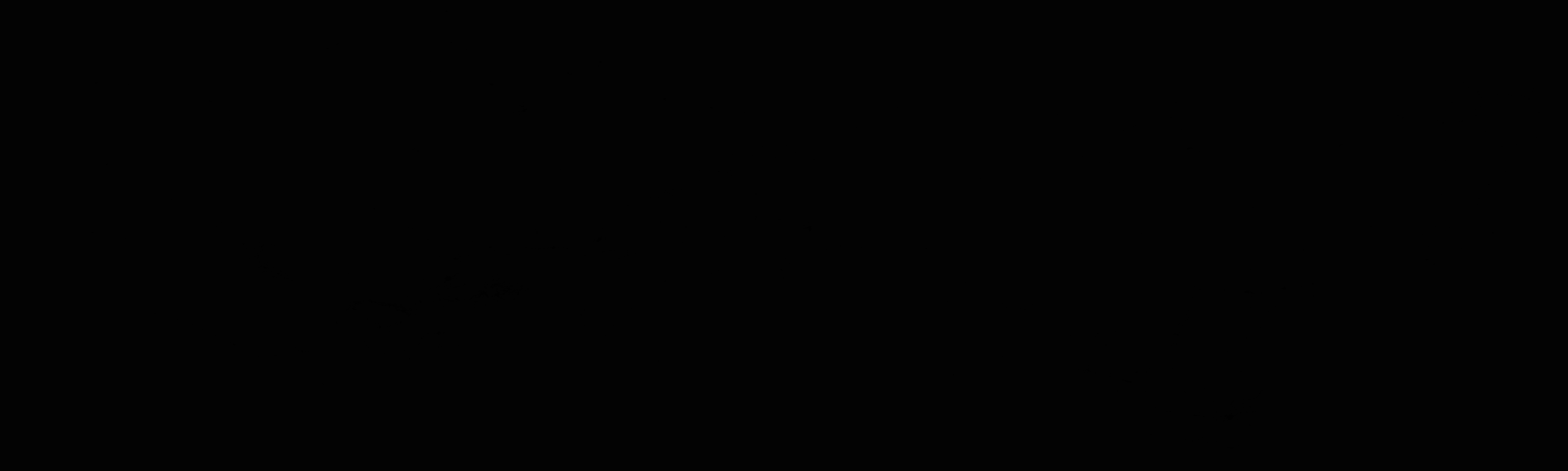

Supplement: S1 Dataset — (ZIP) [file pone.0344835.s001.zip › LUCC2022/UR.tif]

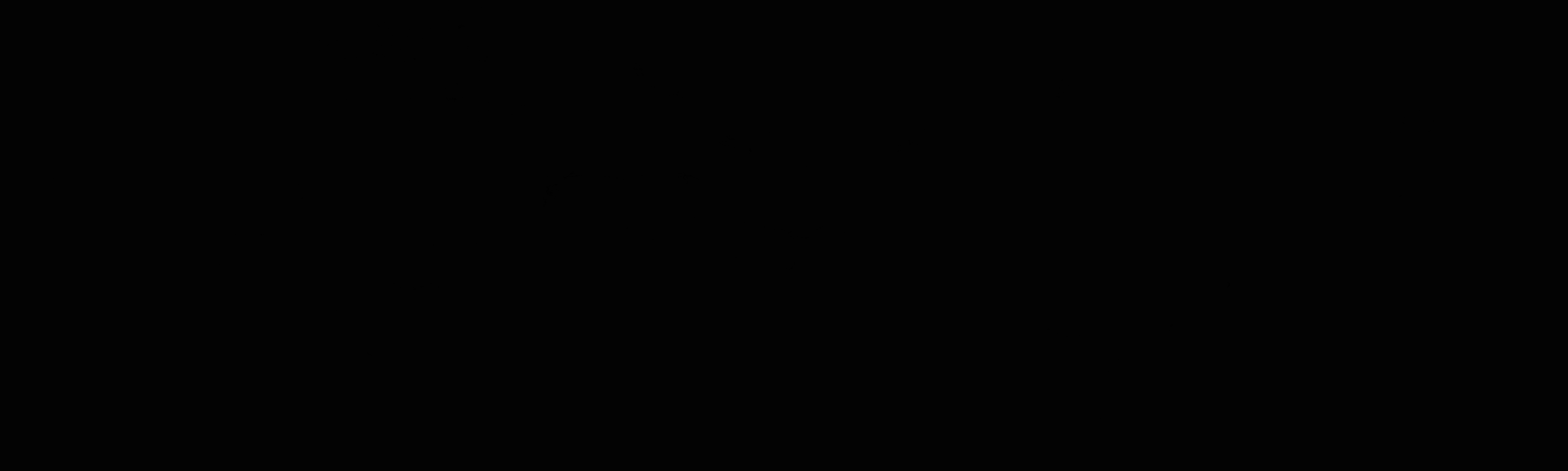

Supplement: S1 Dataset — (ZIP) [file pone.0344835.s001.zip › LUCC2022/WET.tif]

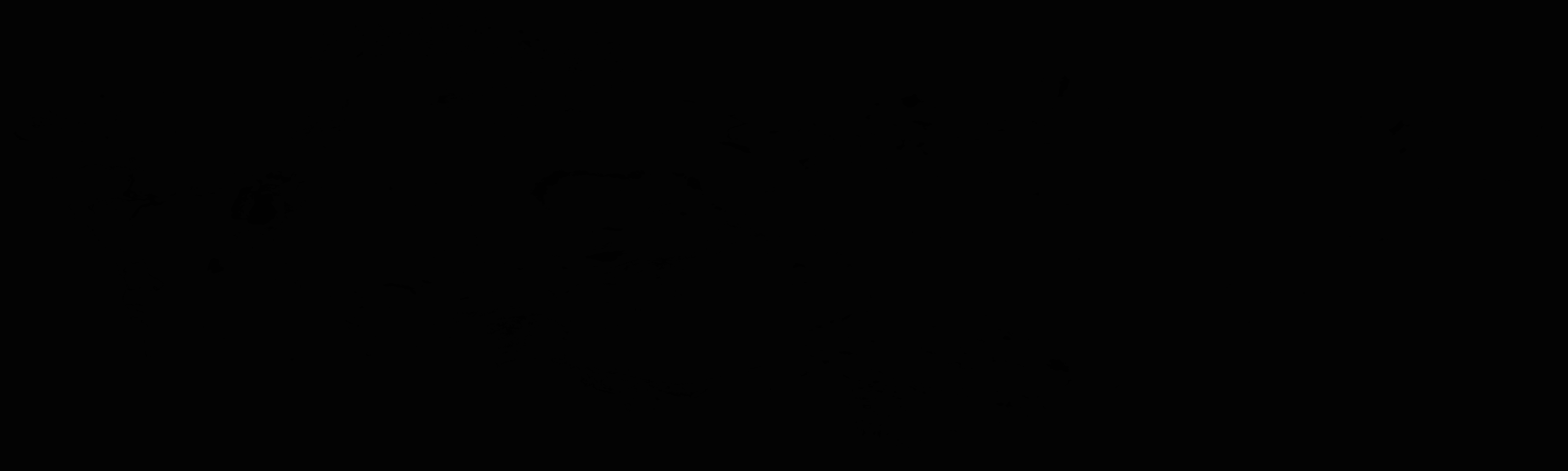

Supplement: S1 Dataset — (ZIP) [file pone.0344835.s001.zip › LUCC2022/WSI.tif]
